# Effects of Chronic Low-Dose Radiation on Human Neural Progenitor Cells

**DOI:** 10.1038/srep20027

**Published:** 2016-01-22

**Authors:** Mari Katsura, Hiromasa Cyou-Nakamine, Qin Zen, Yang Zen, Hiroko Nansai, Shota Amagasa, Yasuharu Kanki, Tsuyoshi Inoue, Kiyomi Kaneki, Akashi Taguchi, Mika Kobayashi, Toshiyuki Kaji, Tatsuhiko Kodama, Kiyoshi Miyagawa, Youichiro Wada, Nobuyoshi Akimitsu, Hideko Sone

**Affiliations:** 1 Isotope Science Center, The University of Tokyo, Tokyo, Japan; 2 Center for Environmental Risk Research, National Institute for Environmental Studies, Tsukuba, Japan; 3 Faculty of Pharmaceutical Sciences, Department of Pharmacy, Tokyo University of Science, Noda, Japan; 4 Research Center for Advanced Science and Technology, The University of Tokyo, Tokyo, Japan; 5 Division of Nephrology and Endocrinology, Graduate School of Medicine, The University of Tokyo, Tokyo, Japan; 6 Laboratory of Molecular Radiology, Center for Disease Biology and Integrative Medicine, Graduate School of Medicine, The University of Tokyo, Japan

## Abstract

The effects of chronic low-dose radiation on human health have not been well established. Recent studies have revealed that neural progenitor cells are present not only in the fetal brain but also in the adult brain. Since immature cells are generally more radiosensitive, here we investigated the effects of chronic low-dose radiation on cultured human neural progenitor cells (hNPCs) derived from embryonic stem cells. Radiation at low doses of 31, 124 and 496 mGy per 72 h was administered to hNPCs. The effects were estimated by gene expression profiling with microarray analysis as well as morphological analysis. Gene expression was dose-dependently changed by radiation. By thirty-one mGy of radiation, inflammatory pathways involving interferon signaling and cell junctions were altered. DNA repair and cell adhesion molecules were affected by 124 mGy of radiation while DNA synthesis, apoptosis, metabolism, and neural differentiation were all affected by 496 mGy of radiation. These *in vitro* results suggest that 496 mGy radiation affects the development of neuronal progenitor cells while altered gene expression was observed at a radiation dose lower than 100 mGy. This study would contribute to the elucidation of the clinical and subclinical phenotypes of impaired neuronal development induced by chronic low-dose radiation.

The effects of low dose radiations on human health have recently attracted considerable attention because of the increasing medical use of ionizing radiation, including interventional radiology and frequent radiological examinations, as well as environmental events, such as atomic power plant accidents^1^. In general, low- and moderate- dose radiations are of less than 100–200 mSv and 500–1000 mSv, respectively. Reports on the atomic bomb survivors of Hiroshima and Nagasaki have shown that cancer risks increase dose-dependently from 150 mGy to 2 Gy^4^ of their absorbed doses. Nevertheless, the effects of lower doses of radiation have not been established. For example, following 50 mGy and 60 mGy of medical exposure to children, increased risks of leukemia and brain tumors, respectively, have been reported^5^. However, many scientists have been suspicious of this finding because of various biases which cannot be excluded in the study. Therefore, it remains to be determined whether less than 100 mGy of radiation exerts an effect on tumorigenesis.

Bergonie–Tribondeau’s law predicts that quickly dividing tumor cells are generally more sensitive to radiation than the majority of quiescent cells. Therefore, irradiations are more effective on cells that have high potential mitotic activity^6^. International Commission on Radiological Protection (ICRP)^7^ has announced that no less than 300 mGy of radiation appears to cause disorders in the fetal brain, and moreover, a dose less than 100 mGy has no effect on IQ. There are several reports showing the effect of less than 300 mGy of radiation on brain development. Among the in utero atomic bomb survivors at the critical embryonic stage of weeks 8–15, severe mental retardation was observed following even a low-dose exposure of 100 mGy^8^,^9^. However, it was difficult to determine the effects of low dose radiation statistically. Furthermore, in mouse models, low-dose radiation of 100 mGy caused neuronal apoptosis in mouse cerebral cortexes on embryonic day 13.5^10^. DNA double strand breaks (DSBs) and apoptosis were induced by low-dose radiation of less than 100 mGy in the ventricular and subventricular zones (VZ/SVZ) on mouse embryonic day 5.5 to 6.5^11^. The biological effects of low-dose irradiation to neuronal development remain unresolved.

Besides the consideration of radiation effects on fetuses, recent studies have revealed that even in the adult brain there are neural progenitor cells in the subgranular zone of the dentate gyrus of the hippocampus as well as SVZ^12^,^13^. Thus, radiation sensitive neural progenitor cells may also exist in the adult brain. If neural cells become injured, such progenitor cells may help them recover their function. Low-dose radiation induces molecular networks and pathways associated with compromised cognitive function in advanced aging and Alzheimer’s disease or dementia within just a few hours of low dose exposure^14^. Moreover, a dose-dependent increase in normal tension glaucoma (NTG) with an odds ratio of 1.31 following irradiations of 1 Gy was reported in atomic bomb survivors^15^. While the mechanism of the increase in NTG by irradiation is unclear, the possible presence of neural progenitor cells in human retina has also been reported^16^. In contrast, in mice, total body 100 mGy X-irradiation did not induce Alzheimer’s disease-like pathogenesis or memory impairment, even after 1 or 2 years^17^. From these reports, the response of neural progenitor cells to low dose radiation should be further determined.

Low dose radiation causes different DNA damage responses in various cells^18^,^19^. In human fibroblasts, radiation doses of more than 10 mGy activate DSB repair systems^20^. When the cells are exposed to radiation at a high dose, the DNA double strand damage signal is transmitted from ataxia telangiectasia mutated (ATM) to p53 and p21^21^. Radiation-induced cell death in the brain is considered to be dependent on the ATM-p53 pathway^22^. Downstream of the signal, p53 exhibits brain region-specific activity that results in different radiation sensitivities^23^. However, while high-dose radiation activates the ATM-p53-p21 pathway, low-dose rate radiation does not transmit the signal from ATM to p53 in murine cells^24^. In another report, the accumulation of MDM2, a ubiquitin ligase that degenerates p53, was induced by continuous radiation in cancer cells^25^. Therefore, p53 does not accumulate in response to low dose radiation. Similarly, in immortalized murine NIH/PG13Luc cells, the expression level of Mdm2 for low-dose-rate gamma rays was higher than that for the high-dose rate; 1 to 100 mGy/h^24^. Taken together, low-dose radiation tends to induce less p53 accumulation owing to the increased ubiquitination by Mdm2, the activities of which are cell-type specific. Thus, the various levels of p53 and sequential divergence of cell cycle phase distribution dependence on cell type-specific responses have been reported^18^,^19^. Moreover, elucidation of the molecular mechanisms underlying cell-specific radiation sensitivity is underway^26^.

hNPCs differentiate from embryonic stem (ES) cells and have been used for the mechanistic study of neurogenesis that takes place in regulating the p53-dependent molecular mechanisms that control neuronal precursor and/or progenitor cell proliferation^27^,^28^. In this study, we have determined the effects of low and moderate doses of chronic radiation on hNPCs at three different doses and dose rates over 72 h. Image analysis and gene expression profiling were utilized to assess the effects of this radiation and to elucidate the types of signaling involved in its effect on neuronal differentiation.

## Results

### Inhibition of neural differentiation and development by low and moderate doses of radiation

**Figure 1:**
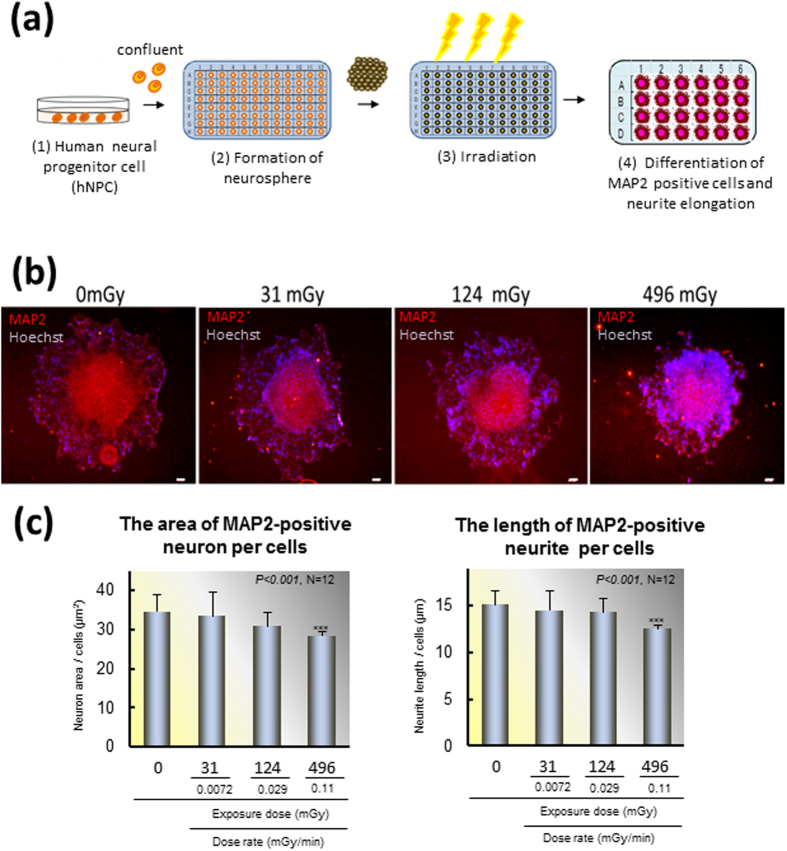
Radiation dose-dependent effect on neurite. hNPCs were exposed to radiation at doses 31, 124, and 496 mGy for 72 h, and neuronal differentiation was analyzed using immunofluorescence. (**a**) Schematic outline of human neural progenitor cell (hNPC) differentiation and the experimental protocol. (1) hNPCs were dispersed and cultivated until they reached confluence in the coated culture plate. (2) Cells were dispersed in a 96-well plate without any coating to form neuropsheres. (3) Each neurospere was exposed to radiation at different doses for 72 hours. (4) After 72 h of radiation, the neurosperes were placed into a coated 24-well plate, and after 6 days they were fixed. (**b**) Immunofluorescence of Microtubule-associated protein 2 (MAP2) and Hoechst. (**c**) Analysis of MAP2 using an IN Cell Analyzer 1000. Left, the area of MAP2-positive neurons per embryoid body (N = 12, ***p < 0.001); Right, the length of MAP2-positive neurites within each embryoid body (N = 12, ***p < 0.001).

To identify the effects on neural differentiation and development associated with radiation exposure, three doses of radiation were applied to hNPCs. Because the formation of neurospheres from hNPCs requires approximately 3 days, we irradiated them for 72 h (Fig. 1a). The total doses of 31, 124 and 496 mGy/72 h correspond to the dose rates of 0.0072, 0.029, and 0.11 mGy/min, respectively. The expression of MAP2, a marker of mature neurons, was examined with immunofluorescence. Neural cell aggregation was caused by irradiation dose-dependently (Fig. 1b). A significant decrease in the area of the neural cells (Fig. 1c, left; N = 12, *p* < 0.001, ANOVA) and neurite length (Fig. 1c, right; N = 12, *p < *0.001, ANOVA) induced by 496 mGy (0.11 mGy/min) of radiation was revealed by computer analysis. However, such effects were not statistically significant following 31 and 124 mGy (0.0072 and 0.029 mGy/min, respectively; Fig. 1c).

### DNA damage response induced by low- and middle-doses of radiation

**Figure 2:**
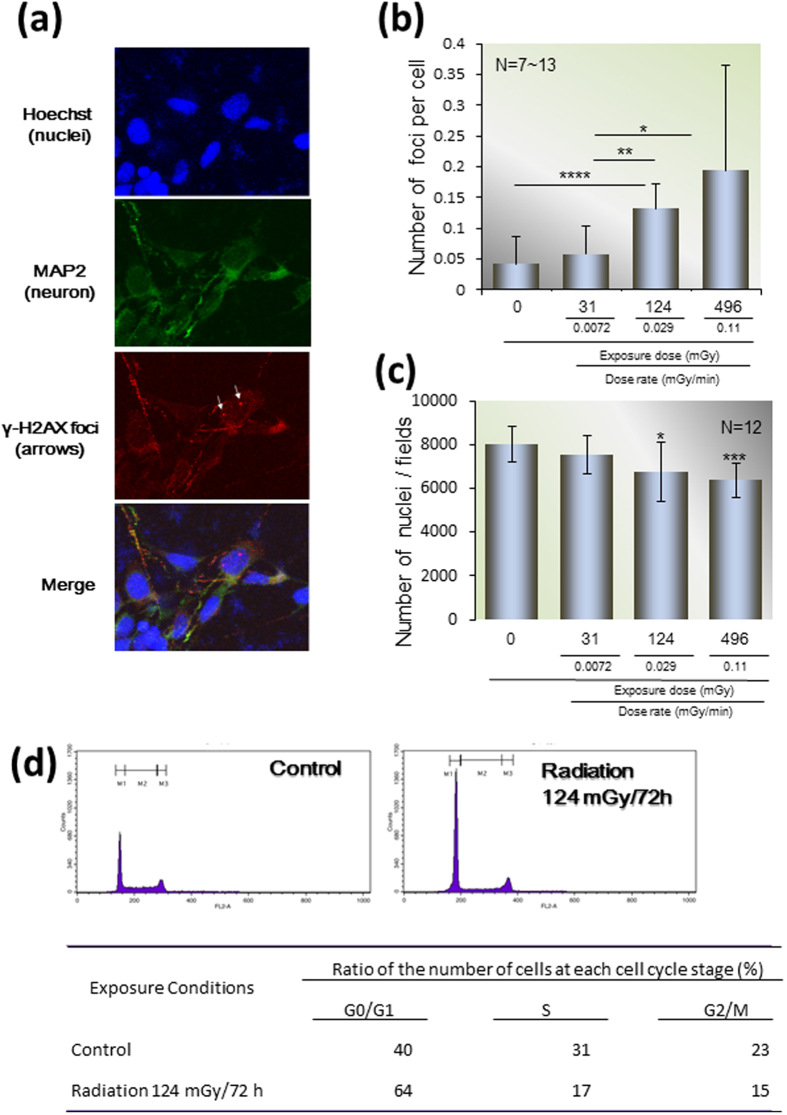
Radiation dose-dependent DNA damage and cell loss in hNPCs. hNPCs were exposed to radiation at doses of 31, 124, and 496 mGy for 72 h, and the DNA damage responses were investigated. (**a**) Nuclei, neurons, and DNA double-strand breaks were visualized by staining with Hoechst, anti-MAP2, and anti-γ-H2AX antibodies, respectively. White arrows indicate γ-H2AX foci in nuclei. Photographs obtained at 60 × magnification. (**b**) The number of nuclear γ-H2AX foci per cell was counted with a confocal microscope using 7–13 biological replicates. (**c**) The number of cells per field was also counted with a confocal microscope. (**d**) Cell cycle analysis after 72 h of irradiation with up to 496 mGy. hNPCs were stained with PI solution and analyzed with a FACS Caliber flow cytometer. (**b,c**) The graph data are the mean ± SD from seven to twelve biological replicates. *p < 0.05, ***p < 0.005, and ****p < 0.0001 by the Student’s *t*-test.

To assess DNA damage, the major effect of radiation, we employed immunofluorescence of γ-H2AX nuclear foci, one of the most commonly used markers of DNA double strand breaks (Fig. 2a). The number of γ-H2AX nuclear foci increased in the three groups from 0.041 ± 0.045 per cell (0 mGy) to 0.057 ± 0.047 (31 mGy, 0.0072 mGy/min), 0.13 ± 0.04 (124 mGy, 0.029 mGy/min), and 0.19 ± 0.17 (496 mGy, 0.11 mGy/min; N = 12, ANOVA, *p* < 0.0001; Fig. 2b) per cell. We counted the number of cells after irradiation. The cell number decreased dose-dependently from 8.0 × 10^3^ ± 8.0 × 10^2^ per field (0 mGy) to 7.5 × 10^3^ ± 8.5 × 10^2^ per field after 31 mGy (0.0072 mGy/min), to 6.7 × 10^3^ ± 1.2 × 10^3^ per field after 124 mGy (0.029 mGy/min; N = 12, *p* < 0.05), and to 6.3 × 10^3^ ± 8.0 × 10^2^ per field after 496 mGy (0.11 mGy/min; N = 12, *p* < 0.005; Fig. 2c). After 72 h of irradiation with 496 mGy, 0.085 mGy per min, the proportion of G0/G1 cells increased from 40% to 64% compared to the non-irradiated cells (Fig. 2d). This indicates that growth arrest was caused by G1 cell cycle checkpoint activation in response to radiation.

### Low- and moderate-dose radiation altered gene expression profiling dose-dependently

**Figure 3:**
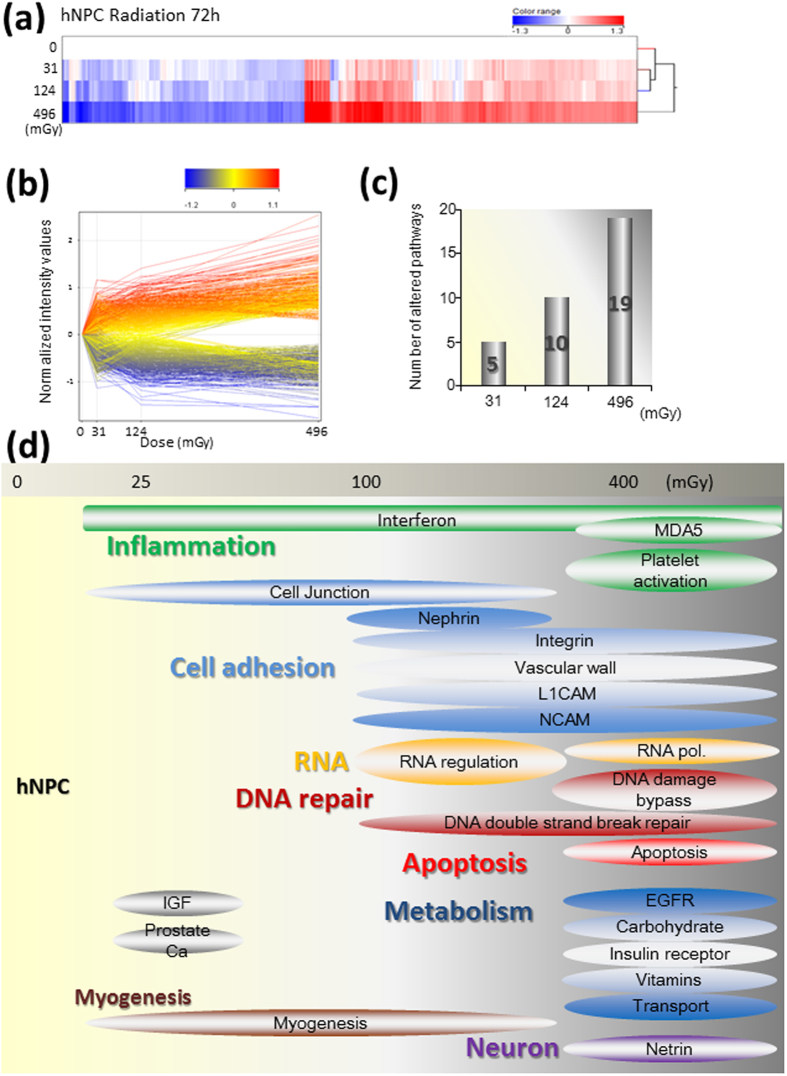
Microarray analysis of radiation dose-dependent gene expression in hNPCs. hNPCs were exposed to radiation at doses 31, 124, and 496 mGy for 72 h, and the collected total RNA was analyzed with an Affymetrix GeneChip.HG-U133 Plus 2 microarray. (**a**) Clustering analysis of genes with more than a 1.5-fold change is shown in a heat-map analyzed by Gene Spring 12.5. (**b**) Fold change analysis by GeneSpring 12.5. The horizontal and vertical axes indicate the radiation dose and normalized intensity values, respectively. (**c**) The number of radiation-dependent altered Wiki pathways analyzed by GeneSpring 12.5. The bars indicate the altered pathways. (**d**) Schematic representation of the dose-dependent alteration of pathways using microarray analysis of hNPCs. The dose-dependently altered pathways detected by GeneSpring 12.5 analysis are shown.

After 72 h of irradiation at the three doses of 31 mGy (0.0072 mGy/min), 124 mGy (0.029 mGy/min), and 496 mGy (0.11 mGy/min), hNPC gene expression was analyzed (Fig. 3). Bioinformatic analysis based on GeneSpring version 12.5 detected a total of 38052 loci sets accurately. Among them, 1035 loci exhibited more than a 1.5-fold change in expression compared to the control as demonstrated in the clustering analysis as well as a heat map (Fig. 3a) and self-organizing map (SOM; Fig. 3b). Both the number of changed loci and fold changes were increased dose-dependently (Fig. 3a,b).

Next, pathway analysis was performed with GeneSpring ver. 12.5 using the Wiki pathway database. The pathways with *p*-values less than 0.05 were five, ten, and nineteen within the 31 mGy (0.0072 mGy/min), 124 mGy (0.029 mGy/min), and 496 mGy (0.11 mGy/min) treatments, respectively (Fig. 3c and Tables 1, 2, and 3). The number of pathways increased dose-dependently (Fig. 3d).

After 31 mGy of radiation (0.0072 mGy/min), the induction of interferons, suppression of cell adhesion, and induction of insulin-like growth factors were detectable, all of which indicate inflammatory responses. In addition, the 124 mGy (0.029 mGy/min) and 496 mGy (0.11 mGy/min) treatments altered five additional pathways related to cell adhesion, nephrin, integrin, the vascular wall, L1CAM and NCAM pathways, which are important for neural function^29^. The DNA DSB repair pathway was activated by 124 mGy (0.029 mGy/min) of radiation. Moreover, the DNA damage bypass pathway, including the DNA polymerase Eta, which is an error-prone translational synthesis (TLS) pathway factor, was activated by 496 mGy (0.11 mGy/min) of radiation. As for RNA, following 124 mGy (0.029 mGy/min) of irradiation, regulation by Dicer was induced, and after 496 mGy (0.11 mGy/min) of irradiation, there were more changes in RNA polymerase, which indicates changes in transcription. Metabolic changes, apoptosis, and neuronal function are also involved in the response to 496 mGy (0.11 mGy/min) of irradiation (Fig. 3d). Netrin is an important axonal guidance factor^30^. Its suppression resulted in a shortening of the axons (Fig. 1b,c). Taken together, the serious effects of 496 mGy (0.11 mGy/min) of irradiation are evidenced by drastic changes in these transcription factor expression levels.

**Figure 4:**
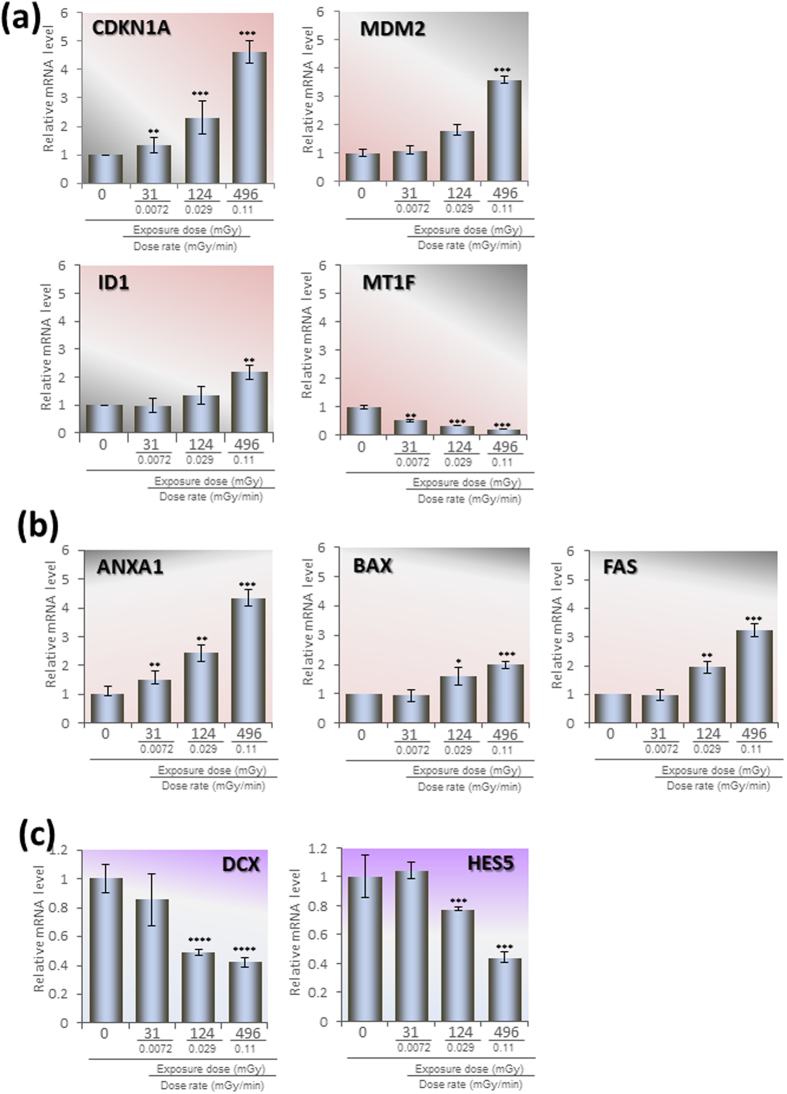
Confirmation of radiation dose-dependent gene expression of hNPCs by qPCR. hNPCs were exposed to radiation at doses 31, 124, and 496 mGy for 72 h, and the collected total RNA was analyzed by qPCR. (**a**) DNA damage response and stress response-related genes. (**b**) Apoptosis-related genes. (**c**) Neural differentiation-related genes. (**a–c)** Quantification was conducted with the *GAPDH* gene. The means ± SD from three independent experiments are shown. *p < 0.05, **p < 0.01, ***p < 0.005, ****p < 0.001

To confirm the fold changes identified by microarray analysis, we performed q-PCR (Fig. 4). First, DNA damage response and stress response factors were determined (Fig. 4a). Cyclin-dependent kinase inhibitor 1A (CDKN1A), also known as p21, one of the most important factors downstream of p53, significantly increased in a dose-dependent manner (*p* < 0.005; Fig. 4a, top left). The MDM2 proto-oncogene (MDM2), one of the E3 ubiquitin ligases targeting p53, also increased dose-dependently (*p* < 0.005; Fig. 4a, top right). The inhibitor of DNA binding 1, a dominant negative helix-loop-helix protein (ID1; Fig. 4a, bottom left), plays a role in cell growth, senescence, and differentiation as a repressor of transcription^31^. While its expression was not changed by the low-dose radiation of 31 mGy (0.0072 mGy/min), moderate-dose radiation of 496 mGy (0.11 mGy/min) increased it significantly (*p* < 0.01; Fig. 4a, bottom left). Metallothionein 1F plays a role in tumor suppression. It was significantly decreased by low- and moderate-dose radiation dose-dependently (*p* < 0.005; Fig. 4a, bottom right).

Certain genes involved in apoptosis were also determined (Fig. 4b). Annexin 1 (ANXA1) plays an important role in anti-inflammatory activity^32^. Its expression was increased by even the low-dose radiation of 31 mGy (0.0072 mGy/min; *p* < 0.01) and more by 124 mGy (0.029 mGy/min; *p* < 0.01) and 496 mGy (0.11 mGy/min; *p* < 0.005; Fig. 4b, left). BCL2-associated X protein (BAX) functions as an apoptotic activator^33^. While its expression was not increased by the low-dose radiation of 31 mGy (0.0072 mGy/min), both 124 mGy (0.029 mGy/min; *p* < 0.05) and 496 mGy (0.11 mGy/min; *p* < 0.005) of irradiation increased it significantly (Fig. 4b, middle). Fas cell surface death receptor (FAS) is a member of the TNF-receptor superfamily, the death domain of which positively regulates apoptosis^34^. The increases in *FAS* by 124 mGy (0.029 mGy/min; *p* < 0.01) and 496 mGy (0.11 mGy/min; *p* < 0.005) of irradiation appeared to induce apoptosis in concert with *BAX* (Fig. 4b, right; *p* < 0.005). Taken together, in addition to the inflammatory reaction induced by 31 mGy (0.0072 mGy/min) of low-dose irradiation, apoptosis-related genes were induced by larger doses of radiation, such as 124 mGy (0.029 mGy/min) and 496 mGy (0.11 mGy/min; Fig. 4b).

Next, we determined the effect on gene expression of certain neural cell-specific molecules (Fig. 4c). Doublecortin (DCX) is an important factor that interacts with microtubules in developing cortical neurons^35^. Thus, it is essential for proper brain function. While the reduction of *DCX* by 31 mGy (0.0072 mGy/min) of irradiation was insignificant, 124 mGy (0.029 mGy/min) and 496 mGy (0.11 mGy/min) of irradiation decreased its expression significantly (*p* < 0.001; Fig. 4c, left). Hes family member bHLH transcription factor 5 (*HES5*) is activated downstream of the Notch pathway. It regulates cell differentiation in multiple tissues^36^. It was also significantly down-regulated by 124 mGy (0.029 mGy/min; *p* < 0.005) and 496 mGy (0.11 mGy/min) of radiation (*p* < 0.005; Fig. 4c, right). Taken together, these two genes involved in hNPC neuronal differentiation were suppressed by 124 mGy (0.029 mGy/min) of radiation (Fig. 4c). All of the pathways with significant alterations and their involved genes in hNPC are shown in Tables 1, 2, and 3 for 31 mGy (0.0072 mGy/min), 124 mGy (0.029 mGy/min), and 496 mGy (0.11 mGy/min), respectively.

To compare these effects to those in another cell line, we examined the gene expression profiles of HUVECs exposed to the same doses of radiation (Supplemental materials, Figure S1, Tables S4, S5, and S6). We found some differences between hNPCs and HUVECs. While in hNPC the alteration of DNA double strand break repair was induced only by more than 124 mGy of radiation and no cell cycle related pathway was changed, in HUVECs the DSB repair pathway and cell cycle pathway were changed by doses of 31 mGy and 124 mGy. This indicates that DNA DSB repair genes are activated under a lower dose of radiation in HUVECs than those in hNPCs.

The microarray data have been deposited in the Gene Expression Omnibus repository (GEO) http://www.ncbi.nlm.nih.gov/geo/, GSE67309^37^.

## Discussion

In this study, we demonstrate the alteration of morphological analysis, including neural differentiation and DNA damage responses, and gene expression in cultured hNPCs by low dose radiation. No tissues are assumed to demonstrate functional impairment due to deterministic effects such as deleterious tissue reactions in the absorbed dose range up to 100 mGy^38^. Severe mental retardation was inferred to be caused by a dose-threshold of at least 300 mGy during the most sensitive pre-natal period (8–15 weeks post-conception). There is also no influence on IQ at intrauterine radiation doses below 100 mGy to the fetus. The purpose of this study was to examine the comprehensive effects of low dose radiation on neural progenitor cells *in vitro*, which may be important for adults as well as fetuses. These cells can be also involved in common neural disorders that are considered to be age-related, such as mild cognitive impairment (MCI) and normal tension glaucoma (NTG). More detailed effects of low- and moderate-dose radiation on neural cells are obviously required to assess the risk of increasing the radiation doses of workers exposed both in accidents and, more generally, in hospitals using advancing medical technologies.

Transcriptional responses to low-dose radiation depend not only on the total absorbed dose but also on the dose rate. In this study, we employed three grades of doses and dose rates, 31 mGy (0.0072 mGy/min), 124 mGy (0.029 mGy/min), and 496 mGy (0.11 mGy/min), indicating that neither the total dose nor the dose rate was fixed. Therefore, the variation of cellular responses among experimental groups reflects both dose and dose-rate effects. The selection of these total doses and dose rates has practical meaning in radiation protection because residents and workers would have a risk of radiation exposure at similar doses and dose rates in nuclear and radiation disasters.

In agreement with ICRP pubulication103, our investigation revealed a significant decrease in the MAP-2 positive cells and neurite length following 496 mGy of irradiation. There was a dose-dependent increase in the γ-H2AX nuclear foci following 124 mGy of radiation. Thirty-one mGy of radiation did not significantly alter DNA DSB repair pathways in hNPCs. Despite the absence of transcriptional changes in DSB repair genes in hNPCs following 31 mGy of irradiation, in HUVECs, the pathway was changed (see Supplemental Material, Figure S1 and Table S1). These findings suggest the presence of cell-type-specific divergence in the DNA damage response to chronic low-dose radiation, consistent with a previous study^18^. Furthermore, the apoptosis pathway was activated in hNPCs by 496 mGy but not in HUVECs at the same dose. Although the survival of these cells was not directly compared in this study, the results suggest that hNPCs are more sensitive to radiation.

G1 cell cycle checkpoint activation was also induced by radiation. The transcription of *CDKN1A* (*p21*) was dose-dependently increased from 31 mGy to 496 mGy. The main factor upstream of CDKN1A is p53, and its activity depends mostly on its phosphorylation status. On the other hand, the stability of p53 is controlled by ubiquitination by the ubiquitin ligase E3 MDM2. Therefore, MDM2 counteracts CDKN1A. *MDM2* was also increased dose-dependently. The finding of an increase in *MDM2* expression by low-dose chronic radiation is consistent with previous studies in both murine cells and human cancer cells^24^,^25^ Considering the dose-dependent increase in the expression of *CDKN1A* and *MDM2*, the activities of p53, which mainly depend on protein modifications, should also change dose-dependently. Under such circumstances, activation of the apoptosis pathway, including ANXA1, FAS, and BAX following 496 mGy of irradiation, may be related to the p53-dependent pathway.

Because the number of γ-H2AX foci reflects the number of DNA DSBs, the dose-dependent increase in the number of γ-H2AX foci was expected. Because the DNA repair pathway, which includes 18 genes involved in DNA DSB repair (Table S4), did not respond to 31 mGy but responded to 124 mGy, γ-H2AX foci were induced by 124 mGy of radiation. The repair pathways include homologous recombination and non-homologous end-joining (NHEJ). Among them, *53BP1* and *XRCC4* were induced significantly by 496 mGy (*p* < 0.05). Because 53BP1 facilitates NHEJ^39^ and XRCC4 functions mainly in the NHEJ pathway^40^, the alteration of these two molecules indicates that the DNA DSBs were mainly repaired by NHEJ. Furthermore, radiation at 496 mGy induced the DNA damage bypass pathway, which activates TLS mediated by DNA polymerase Eta, HREV7, and HREV3. Because DNA damage repair bypass by TLS is error-prone, radiation of 496 mGy has the potential to cause mutations^41^.

Radiation at levels 124 mGy and 496 mGy altered gene expression of certain molecules involved in apoptosis. Moreover, 496 mGy of irradiation induced an alteration in the intrinsic apoptosis pathway, which acts through the activation of caspase-9 via Apaf-1 and cytochrome c^42^. However, not all of the genes were altered synchronously. In other words, both apoptotic and anti-apoptotic effects were activated by low- and moderate-dose radiation treatments. Considering the gradual decreases in the number of cells during 124 mGy and 496 mGy of irradiation, how the balance between these effects is controlled under chronic radiation is a subject of interest.

Irradiation of 124 mGy also caused transcriptional changes of genes involved in cell adhesion. In anchorage-dependent cells, detaching from the surrounding extracellular matrix induces a process of programmed cell death that is called anoikis^18^,^43^. The nephrin^44^, integrin, L1CAM, NCAM, and vascular wall pathways are all important for nerve differentiation and avoidance of anoikis^43^. Nephrins and integrins are essential for adhesion. In addition, L1CAM and NCAM are crucial for axonal extension and nerve function^43^. Moreover, the metabolism pathways were significantly affected by 496 mGy of radiation. The carbohydrate, insulin, vitamin, and transport pathways were changed (*p* < 0.05). Netrin is essential for axon guidance during neuronal differentiation^45^; when its transcription is altered, neuronal hypo-function is likely to occur.

Present results revealed that the alteration of expression profiling depended on cell types. Although cell-type-specific DNA damage responses, including cell survival, nuclear foci, and the ATM-p53-p21 pathway, have already been reported^18^, to the best of our knowledge this is the first genome-wide, cell-type-specific expression profile suggesting these responses. These results establish the importance of cell-type-specific DNA damage responses under low-dose radiation.

The reference level for radiological emergency is 20 to 100 mSv per year according to ICRP recommendations. Although these dose limits are generally adequate, a determination of more precise effects will contribute to an improved understanding of low-dose radiation effects and the mechanisms underlying its impact on neural differentiation and age-related disorders. Neural progenitor cell damage causes age-related cognitive disorders and/or dementia in some cases^46^. In our experiment, 31 mGy of radiation induced alterations in the interferon and cell junction pathways. From an optimistic point of view, no serious effects resulted from this dose. However, continuation of this same dose rate for a longer period of time may result in further changes, especially in persons with certain genetic or epigenetic conditions and/or in persons harboring few neural progenitor cells. More than 124 mGy (0.029 mGy/min) of radiation may dose-dependently induce certain serious neural disorders. These findings are in agreement with the acceptable dose-level set by the ICRP recommendation. However, according to the improvement in information systems and the accumulation of the biological knowledge, genetic or epigenetic causes of individual variation in sensitivity to radiation will be well-defined, in future. From this perspective, it is of critical importance to elucidate the molecular events that underlie changes in the neural progenitor cells induced by low- to moderate-dose radiation exposure.

This paper demonstrates that morphological and transcriptional alteration of hNPCs is induced by chronic radiation. Thirty-one mGy (0.0072 mGy/min) of irradiation caused a slight alteration in the inflammatory gene expression. Doses of 124 mGy (0.029 mGy/min) or more for 72 h of radiation may promote the onset of neural disorders, and this effect appears to be related to the p53-dependent DNA damage response. Potentials of the low dose radiation effects in neural progenitor cells should be further clarified.

## Materials and Methods

### Cell Culture

This study used an hNPC line (ENStem-A Human Neural Progenitor Expansion Kit; SCR055) obtained from Millipore. hNPCs were seeded at a density of 2 × 10^6^ cells/20.8 cm^2^ for growth and maintenance [Fig. 1a (1) Proliferation of human neural progenitor cells (hNPCs)]. hNPCs were seeded at a density of 2 × 10^6^ cells/20.8 cm^2^ for growth and maintenance as reported previously^47^,^48^. After the cells reached confluence, they were dissociated with accutase dissociation solution (ICT, FUAT104) and then transferred into a 96-well U-bottom plate (Nunc™ low cell binding plate, Cat No. 145399, Thermo Fisher Scientific Inc. Waltham, MA, USA) at a density of 6 × 10^3^ cells/well for 2 days to form a neurosphere (2). Each neurospere was exposed to radiation at different doses for 72 hours (3). After exposure, NPSs were transferred and seeded onto another 24-well plate precoated with poly-ornithine–laminin 511 (poly-L-ornithine P4957, Sigma-Aldrich; LN511, No. BLA-LN511-03, BioLamina AB, Stockholm, Sweden) to promote neuronal differentiation by the sequential exchange of neuronal proliferation media to differentiation media (4). Human umbilical vein endothelial cells (HUVECs) from the sixth to twelfth passages were grown in endothelial cell basal medium-2 (EBM-2; Lonza, Basel, Switzerland) with 5% fetal bovine serum (FBS). After confluence, the medium was replaced with one containing 0.5% FBS to synchronize the cells for 16 h. Then they were exposed to radiation and subsequently used for microarray analysis. All experiments were approved by the ethics committees of the University of Tokyo and National Institute for Environmental Studies in accordance with the guideline of the Japanese Ministry of Education, Culture, Sports, Science, and Technology.

### Radiation exposure

A sealed radiation source of 18.5 GBq Cs-137 (CsCl) was used for irradiation to the cells in a carbon dioxide (CO_2_) incubator for 72 h. The doses for external-exposure were 0 mGy, 31 mGy (0.0072 mGy/min), 124 mGy (0.029 mGy/min), and 496 mGy (0.11 mGy/min), which were controlled by the distance between the cell cultures and radiation source. The doses of radiation were measured with a fluoroglass dosimeter (RPL Glass Dosimetery, AGC Tecno Glass Co., Ltd., Funabashi, Japan) placed under each plastic culture plate that was analyzed with an automatic reader (FGD-202, Toshiba, Tokyo, Japan). Within the 72 h of radiation, the hNPCs had differentiated into neuronal cells. Then the neuronal cells were immediately fixed for immunofluorescence.

### Immunofluorescence

After fixation for 15 min with 4% paraformaldehyde at room temperature, the cells were permeabilized for 30 min in 0.1% Triton X100 with 5% goat serum diluted in phosphate-buffered saline (PBS). Then, the cells were incubated with 1% BSA, which was followed by overnight incubation with a primary antibody specific to MAP2 (1:200; Sigma-Aldrich, St. Louis, MO, USA) and γ-H2AX (1:400; Cell Signaling Technology, Beverly, MA, USA). After washing with PBS, the cells were incubated at room temperature for 1 h with Alexa 488-labeled anti-mouse secondary antibody (1:1000; Molecular Probes, Carlsbad CA, USA) and Alexa 546-labeled anti-rabbit secondary antibody (1:1000; Molecular Probes). Nuclei were stained with 2 μg/ml Hoechst 33342 (Sigma-Aldrich) for 15 min.

### Morphological measurement of neural cells

Microphotographs were obtained with an inverted microscope (IX70-22FL/PH; Olympus Co. Tokyo, Japan). Morphological analysis was performed with an automatic multichannel imaging analyzer (IN Cell Analyzer (ICA) 1000; GE healthcare Inc, Buckinghamshire, UK). Photographs of γ-H2AX foci-positive cells were acquired with a z-stack using a confocal laser scanning microscope (FV1200; Olympus).

### Cell cycle analysis

After the external exposure in a CO_2_ incubator for 72 h, hNPCs were trypsinized, resuspended in fresh media, and stained with propidium iodide (BD Biosciences, East Rutherford, NJ, USA) according to the manufacturer’s instructions. Then cells were read on a BD FACS Calibur flow cytometer (BD Biosciences). Twenty-thousand cells were analyzed for each sample (Fig. 2c).

### Expression array profiling and analysis

Total RNA from the hNPCs was collected just after 72 h of irradiation at levels up to 496 mGy (0.085 mGy/min) and purified using an RNeasy Micro Kit (Qiagen, Tokyo, Japan) in accordance with the manufacturer’s instructions. Quantification and quality assessment of the isolated RNA samples were performed and verified using an Agilent Bio Analyzer 2100 and an RNA 6000 Pico Assay (Agilent Technologies, Santa Clara, CA) in accordance with the manufacturer’s instructions. All the samples were profiled on an Affymetrix GeneChip® Human Genome U133 Plus 2.0 Array (Affymetrix, San Diego, CA). The expression value for each mRNA was obtained using the Robust Multi-array Analysis (RMA) method. After excluding the gene set probes which did not have gene symbols, the remaining loci were used for further analysis. The probes, which were expressed at <20 percentile in all of the seven arrays, were eliminated from the analyses using GeneSpring 12.5 (Agilent Technologies) and filtered for genes with a greater than 1.5-fold change. Heat maps of selected gene lists, and pathway analyses were also performed with GeneSpring 12.5. Hierarchical clustering analysis was performed using average linkages and Pearson correlations as a measure of similarity. Annotation of the probe numbers and target sequences are shown on the Affymetrix web page.

### Quantitative Real Time PCR (qPCR)

Gene expression of radiation sensitive genes in hNPCs was investigated by qPCR performed using the SYBR^®^ Green PCR Master Mix (Takara Bio, Ohtsu, Japan) according to the manufacturer’s instructions. The amplification reaction was performed in the Thermal Cycler Dice^®^ Real Time System Single (Takara Bio) under the following cycling conditions, one cycle of 95 °C for 30 s followed by 40 cycles of 95 °C for 15 s and 60 °C for 30 s. The triplicated gene expressions were normalized by the *GAPDH* expression of the control. The PCR primers were as follows: *CDKN1A* Forward tcactgtcttgtacccttgtgc, Reverse ggcgtttggagtggtagaaa; *MDM2* Forward catgcctgcccactttaga, Reverse ggaggctcccaactgctt; *ID1* Forward ccagaaccgcaaggtgag, Reverse ggtccctgatgtagtcgatga; *MT-1F* Forward cgtgggctgtagcaagtgt, Reverse aaaggttgtcctggcatcag; *ANXA1* Forward cagtaagcatgacatgaacaaagtt, Reverse gaaagctggtttgcttgtgg; *BAX* Forward atgttttctgacggcaacttc, Reverse atcagttccggcaccttg; *FAS* Forward atggccaattctgccataag, Reverse tgactgtgcagtccctagctt; *DCX* Forward aggatgcctatatccccaaaa, Reverse tctgaggtggggtgatcttt; *HES5* Forward tcagctacctgaagcacagc, Reverse tagtcctggtgcaggctctt; and *GAPDH* Forward gcaccgtcaaggctgagaac, Reverse tggtgaagacgccagtgga.

### Statistical analysis

The data are presented as the mean ± standard deviation (SD) of at least three independent experiments. Statistical significance was determined by the Student’s *t*-test for two group comparisons between the treatments and the control. Additional analyses were performed by one-way ANOVA followed by Dunnett’s tests for pairwise comparisons. These differences were considered statistically significant when *P* < 0.05.

## Additional Information

**Accession codes:** GEO GSE67309, Katsura, M., Low- and middle-dose of radiation on hNPC and HUVEC (2015) (Date of access: Sep 14, 2015).

**How to cite this article**: Katsura, M. *et al.* Effects of Chronic Low-Dose Radiation on Human Neural Progenitor Cells. *Sci. Rep.* **6**, 20027; doi: 10.1038/srep20027 (2016).

## Supplementary Material

Supplementary Information

## Figures and Tables

**Table 1:** Pathways changed in hNPCs by 31 mGy of radiation.

Pathways changed by 31 mGy of radiation	*p*-value	Matched Entities	Pathway Entities of Experiment Type
Hs_Interferon_alpha-beta_signaling_WP1835_44864	0.006196	2	26
Hs_Regulation_of_Insulin-like_Growth_Factor_(IGF)_Activity_by_Insulin-like_Growth_Factor_Binding_Proteins_(IGFBPs)_WP1899_45051	0.048129	1	10
Hs_Cell_junction_organization_WP1793_44989	2.50E-04	3	28
Hs_Myogenesis_WP1865_44931	0.02436	1	6
Hs_Prostate_Cancer_WP2263_69730	0.00192	4	116

**Table 2:** Pathways changed in hNPCs by 124 mGy of radiation.

Pathways changed by 125 mGy of radiation	*p*-value	Matched Entities	Pathway Entities of Experiment Type
Hs_Double-Strand_Break_Repair_WP1807_45201	0.008841	2	18
Hs_Integrin_cell_surface_interactions_WP1833_44861	0.00537	2	16
Hs_L1CAM_interactions_WP1843_44884	9.20E-07	5	27
Hs_Regulatory_RNA_pathways_WP1901_45048	0.015812	1	2
Hs_Interferon_alpha-beta_signaling_WP1835_44864	3.67E-05	4	26
Hs_Cell_surface_interactions_at_the_vascular_wall_WP1794_42017	0.029829	2	39
Hs_Nephrin_interactions_WP1867_42085	9.23E-04	2	7
Hs_Cell_junction_organization_WP1793_44989	0.016714	2	28
Hs_Myogenesis_WP1865_44931	0.039064	1	6
Hs_NCAM_signaling_for_neurite_out-growth_WP1866_42084	7.81E-05	3	13

**Table 3:** Pathways changed in hNPCs by 496 mGy of radiation.

Pathways changed by 496 mGy of radiation	*p*-value	Matched Entities	Pathway Entities of Experiment Type
Hs_Double-Strand_Break_Repair_WP1807_45201	0.034379	2	18
Hs_Integrin_cell_surface_interactions_WP1833_44861	0.001387	3	16
Hs_Intrinsic_Pathway_for_Apoptosis_WP1841_44875	2.79E-04	4	21
Hs_Transport_of_glucose_and_other_sugars,_bile_salts_and_organic_acids,_metal_ions_and_amine_compounds_WP1935_45063	0.007261	4	48
Hs_L1CAM_interactions_WP1843_44884	3.05E-05	5	27
Hs_Interferon_alpha-beta_signaling_WP1835_44864	5.82E-04	4	26
Hs_Signaling_by_EGFR_WP1910_45218	0.038015	2	21
Hs_RIG-I-MDA5_mediated_induction_of_IFN-alpha-beta_pathways_WP1904_45045	0.045699	2	21
Hs_Cell_surface_interactions_at_the_vascular_wall_WP1794_42017	0.002231	4	39
Hs_Metabolism_of_carbohydrates_WP1848_44895	0.030888	2	17
Hs_Signaling_by_Insulin_receptor_WP1913_45215	0.038015	2	21
Hs_NCAM_signaling_for_neurite_out-growth_WP1866_42084	6.52E-04	3	13
Hs_DNA_Damage_Bypass_WP1803_45195	0.048269	1	3
Hs_RNA_Polymerase_II_Transcription_WP1906_45042	0.003474	3	19
Hs_Platelet_activation_triggers_WP1881_42097	0.032443	1	2
Hs_Netrin-1_signaling_WP1868_42086	0.002498	3	18
Hs_Metabolism_of_water-soluble_vitamins_and_cofactors_WP1857_44904	0.021344	2	16
Hs_Prostate_Cancer_WP2263_69730	0.030416	5	116
Hs_Double-Strand_Break_Repair_WP1807_45201	0.034379	2	18
