# Supplementary material for: Effects of Chronic Low-Dose Radiation on Human Neural Progenitor Cells
Source: Sci Rep. 2016 Jan 22;6:20027. doi: 10.1038/srep20027 (PMC4726121; doi:10.1038/srep20027)
Supplement: Supplementary Information [file srep20027-s1.pdf]

Supplementary information

Title: Effects of Chronic Low-Dose Radiation on Human Neural Progenitor Cells

Running title: Radiation-induced expression profile of hNPCs

Mari Katsura[1], Hiromasa Cyou-Nakamine[1,2,3], Qin Zen[2], Yang Zen[2], Hiroko Nansai[2], Shota Amagasa[1], Yasuharu Kanki[1], Tsuyoshi Inoue[4,5], Kiyomi Kaneki[4], Akashi Taguchi[1,4], Mika Kobayashi[1,4], Toshiyuki Kaji[3], Tatsuhiko Kodama[1,4], Kiyoshi Miyagawa[6], Youichiro Wada[1,4], Nobuyoshi Akimitsu [1], Hideko Sone [2]

1 Isotope Science Center, The University of Tokyo, Tokyo, Japan;
2 Center for Environmental Risk Research, National Institute for Environmental Studies, Tsukuba, Japan;
3 Faculty of Pharmaceutical Sciences, Department of Pharmacy, Tokyo University of Science, Noda, Japan;
4 Research Center for Advanced Science and Technology, The University of Tokyo, Tokyo, Japan;
5 Division of Nephrology and Endocrinology, Graduate School of Medicine, The University of Tokyo, Tokyo, Japan;
6 Laboratory of Molecular Radiology, Center for Disease Biology and Integrative Medicine, Graduate School of Medicine, The University of Tokyo

Equal contribution to this work: Mari Katsura and Hiromasa Cyou-Nakamine
Address correspondence to Hideko Sone, Center for Environmental Risk Research, National Institute for Environmental Studies, Tsukuba, Japan, 305-8506 Japan. Telephone: +81 29 850 2464, e-mail: hsone@nies.go.jp

Suppl. Figure 1

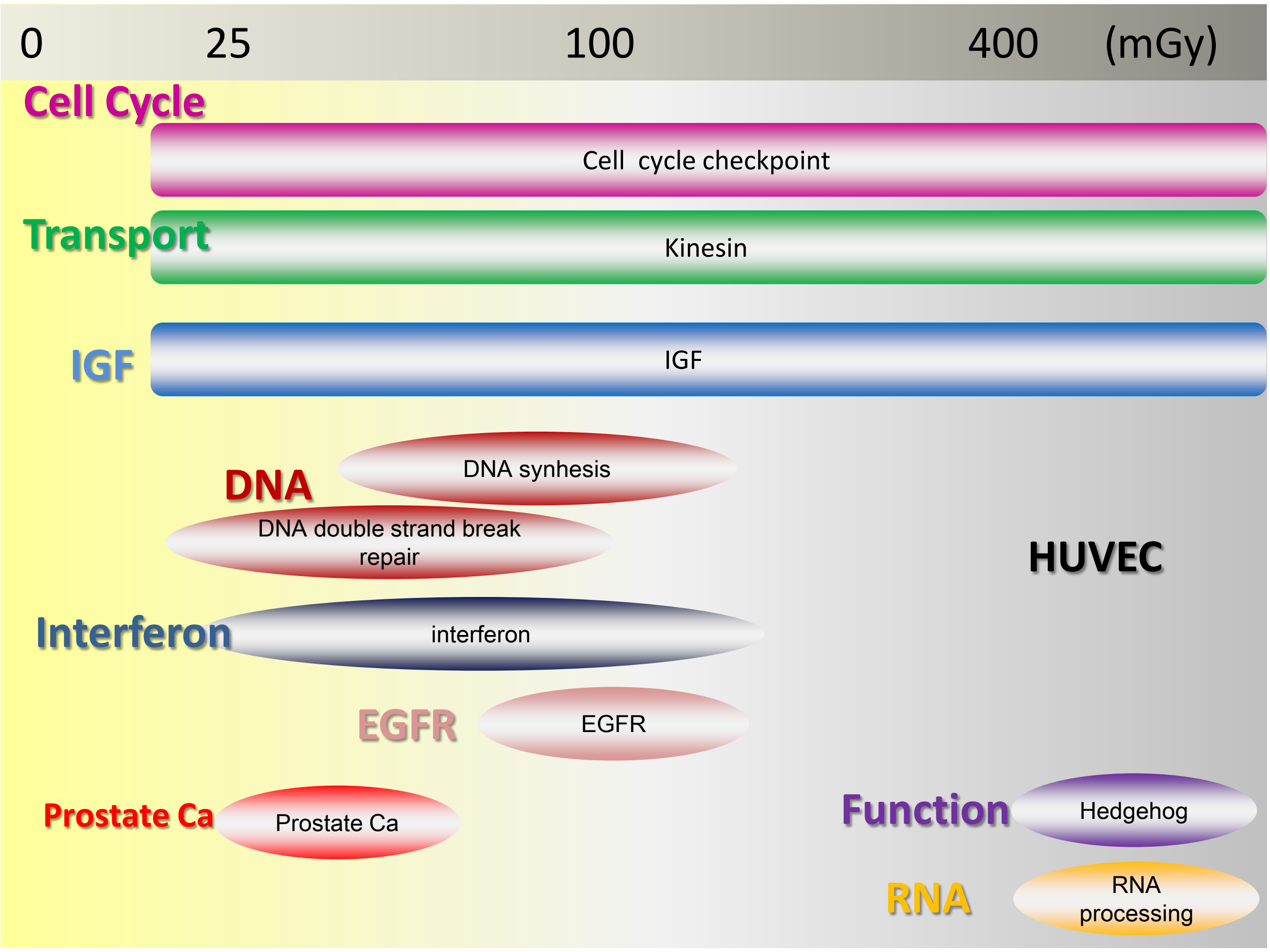

Altered pathways in HUVEC after 72 h of radiation
Wiki pathway IDs are shown in Supplementary Figure 3

HUVECs (DS Pharma Biomedical, Osaka, Japan) from the sixth to tenth passages were grown in endothelial cell basal medium-2 (EBM-2) (Lonza^{R}). After they reached confluence in EBM-2 with 5% FBS, the culture medium was replaced with serum starvation medium with 0.5% FBS for sixteen hours to synchronize the cells in the G1 cell cycle phase before radiation. During the radiation, the concentration of 0.5 % FBS was continued. A sealed radiation source of 18.5 GBq Cs-137 (CsCl) was used for external-exposure exposed in a CO_2 incubator for 72 h. The doses for external-exposure were 0 mGy, 31 mGy (0.0072 mGy/ min), 124 mGy (0.029 mGy/ min) and 496 mGy (0.085 mGy/ min). Microarray analysis was performed in the same way of hNPC.

Suppl. Figure 2

hNPC

31 mGy	124 mGy	496 mGy
Hs_Regulation_of_Insulin-like_Growth_Factor_(IGF)_Activity_by_Insulin-like_Growth_Factor_Binding_Proteins_(IGFBPs)_WP1899_45051		
Hs_Cell_junction_organization_WP1793_44989	Hs_Cell_junction_organization_WP1793_44989	
Hs_Myogenesis_WP1865_44931	Hs_Myogenesis_WP1865_44931	
Hs_Prostate_Cancer_WP2263_69730		Hs_Prostate_Cancer_WP2263_69730
Hs_Interferon_alpha-beta_signaling_WP1835_44864	Hs_Interferon_alpha-beta_signaling_WP1835_44864	Hs_Interferon_alpha-beta_signaling_WP1835_44864
	Hs_Integrin_cell_surface_interactions_WP1833_44861	Hs_Integrin_cell_surface_interactions_WP1833_44861
	Hs_Cell_surface_interactions_at_the_vascular_wall_WP1794_42017	Hs_Cell_surface_interactions_at_the_vascular_wall_WP1794_42017
	Hs_L1CAM_interactions_WP1843_44884	Hs_L1CAM_interactions_WP1843_44884
	Hs_NCAM_signaling_for_neurite_out-growth_WP1866_42084	Hs_NCAM_signaling_for_neurite_out-growth_WP1866_42084
	Hs_Double-Strand_Break_Repair_WP1807_45201	Hs_Double-Strand_Break_Repair_WP1807_45201
	Hs_Nephrin_interactions_WP1867_42085	Hs_Netrin-1_signaling_WP1868_42086
	Hs_Regulatory_RNA_pathways_WP1901_45048	Hs_RNA_Polymerase_II_Transcription_WP1906_45042
		Hs_RIG-I-MDA5_mediated_induction_of_IFN-alpha-beta_pathways_WP1904_45045
		Hs_DNA_Damage_Bypass_WP1803_45195
		Hs_Metabolism_of_carbohydrates_WP1848_44895
		Hs_Transport_of_glucose_and_other_sugars,_bile_salts_and_organic_acids,_metal_ions_and_amine_compounds_WP1935_45063
		Hs_Signaling_by_Insulin_receptor_WP1913_45215
		Hs_Platelet_activation_triggers_WP1881_42097
		Hs_Metabolism_of_water-soluble_vitamins_and_cofactors_WP1857_44904
		Hs_Signaling_by_EGFR_WP1910_45218
		Hs_Intrinsic_Pathway_for_Apoptosis_WP1841_44875

HUVEC

31 mGy	124 mGy	496 mGy
Hs APC-C-mediated degeneration of cell cycle proteins WP1782 44955	Hs/APC-C-mediated degradation of cell cycle proteins WP1782-44955	Hs/APC-C-mediated degradation of cell cycle proteins WP1782-44955
Hs Mitotic M-M-G1 phases WP1860 44914	HsMitosis M-M-G1phases WP1860_44914	HsMitosis M-M-G1phases WP1860_44914
Hs Kinesines WP1842 44882	Hs Kinesines WP1842 44882	Hs Kinesines WP1842 44882
Hs Regulation of Insulin like Growth Factor (IGF) Activity by IGF Binding Proteins (IGFBPs) WP1899 45051	Hs Regulation of Insulin like Growth Factor (IGF) Activity by IGF Binding Proteins (IGFBPs) WP1899 45051	Hs Regulation of Insulin like Growth Factor (IGF) Activity by IGF Binding Proteins (IGFBPs) WP1899 45051
Hs Synthesis of DNA WP1925 45107	Hs Synthesis of DNA WP1925_45107	
Hs Interferon alpha-beta signaling WP1835_44864	Hs Interferon alpha-beta signaling WP1835_44864	
Hs Double -Strand Break Repair WP1807_45201		
	Hs_Signaling_by_EGFR_WP1910_45218<br>Signaling by TGFBR Complex_WP2742	
		Hedgehog_Signaling_Pathway_WP47<br>Hs_Phase1-Functionalization of compounds_WP11879_42095<br>Hs Procession of Capped Intron-Containing Pre-mRNA WP1889_42105

HUVEC

Table S1. Altered Wiki pathway IDs in HUVEC after 72 h of radiation at 31 mGy

Function	Pathway Name	p-value	Matched Entitles	Pathway Entitles
31mGy Up				
cell cycle checkpoint	Hs APC-C-mediated degeneration of cell cycle proteins WP1782 44955	1.2E-12	7	10
mitosis	Hs Mitotic M-M-G1 phases WP1860 44914	9.2E-11	7	15
transport	Hs Kinesines WP1842 44882	8.3E-06	3	9
IGF related signaling	Hs Regulation of Insulin like Growth Factor (IGF) Activity by IGF Binding Proteins (IGFBPs) WP1899 45051	0.00012	3	10
S phase DNA replication	Hs Synthesis of DNA WP1925 45107	0.00021	3	13
DNA repair	Hs Double -Strand Break Repair WP1807_45201	7.6E-0.4	3	18
		total	26	
31mGy Down				
Immuno reaction	Hs Interferon alpha-beta signaling WP1835_44864	4.0E-0.4	3	26
		total	3	

HUVEC

Suppl. Table 2

Table S2. Altered Wiki pathway and IDs in HUVEC after 72 h of radiation at 124mGy

Function	Pathway Name	p-value	Matched Entitles	Pathway Entitles
124mGy Up				
Cell cycle checkpoint	Hs/APC-C-mediated degradation of cell cycle proteins WP1782-44955	1.60E-10	6	10
Mitosis	HsMitosis M-M-G1phases WP1860_44914	3.70E-09	6	15
S phase DNA replication	Hs Synthesis of DNA WP1925_45107	6.20E-08	5	13
Transport	Hs Kinesines WP1842 44882	7.20E-05	3	9
Immuno reaction	Hs Interferon alpha-beta signaling WP1835_44864	7.90E-05	4	26
IGF related signaling	Hs Regulation of Insulin like Growth Factor (IGF) Activity by IGF Binding Proteins (IGFBPs) WP1899 45051	1.00E-04	3	10
Growth, Differentiation	Signaling by TGFBR complex WP2742	3.30E-04	7	134
Proliferation	Hs Signaling by EGFR_WP1910_45218	9.10E-04	3	20
		total	37	
124mGy Down				
Growth, Differentiation	Signaling by TGFBR complex WP2742	6.50E-05	8	134
Proliferation	Hs Signaling by EGFR_WP1910_45218	4.20E-04	8	176
		total	16	

Table S3. Altered Wiki pathway and IDs in HUVEC after 72 h of radiation at 496 mGy

Function	Pathway Name	p-value	Matched Entitles	Pathway Entitles
496mGy Up				
cell cycle checkpoint	Hs/APC-C-mediated degradation of cell cycle proteins WP1782-44955	3.10E-11	6	1.00E+01
transport	Hs Kinesines WP1842 44882	3.20E-05	3	9.00E+00
IGF related signaling	Hs Regulation of Insulin like Growth Factor (IGF) Activity by IGF Binding Proteins (IGFBPs) WP1899 45051	4.60E-05	3	1.00E+01
mitosis	HsMitosis M-M-G1phases WP1860_44914	1.70E-04	3	1.50E+01
	Hs_Phase1-Functionalization of compounds_WP11879_42095	4.00E-04	4	4.90E+01
Development	Hs_Hedgehog_Signaling Pathway_WP47	4.10E-04	3	2.00E+01
		total	22	
496mGy Down				
RNA	Hs Procession of Capped Intron-Containing Pre-mRNA WP1889_42105	2.20E-05	5	5.20E+01
		total	5	

Suppl. Table 4. DNA double strand break repair genes classified by Wiki pathway

Hs_Double-Strand_Break_Repair WP1807_45201
phospho-ATM (Ser 1981)
Histone H2A.x/gamma-H2AX
BRCA1/phospho-BRCA1
MDC1/NFBD1/phospho- MDC1/NFBD1
NBS1/phospho-NBS1
53BP1
MRE11A
RAD50
FANCJ/BRIP1
RAD51
BRCA2
RAD52
DNA-dependent protein kinase catalytic subunit/phospho-DNA-PKcs
DNA ligase I
ATP-dependent DNA helicase II, 80 kDa subunit/KU80
ATP-dependent DNA helicase II, 70 kDa subunit/KU70
DNA ligase IV
DNA repair protein XRCC4
